# Carbon Fibers from PAN/PVP Blends by Solution Blow Spinning to Suppress Hydrogen Evolution in Lead-Acid Batteries

**DOI:** 10.1021/acsomega.4c10531

**Published:** 2025-04-22

**Authors:** Caio M. S. Lopes, Juan P. S. Cruz, Rafael A. Raimundo, Vinícius D. Silva, Rogério T. Ribeiro, Daniel A. Macedo, Eudésio O. Vilar, Gilberto A. O. Brito, Eliton S. Medeiros

**Affiliations:** † Department of Materials Science and Engineering, UFPB, João Pessoa 58051-900, Brazil; ‡ Materials and Biosystems Laboratory (LAMAB), DEMAT, UFPB, João Pessoa 58051-900, Brazil; § TEMA - Centre for Mechanical Technology and Automation, Department of Mechanical Engineering, University of Aveiro, Aveiro 3810-193, Portugal; ∥ Electrochemical Eng. Laboratory (LEEQ), Federal University of Campina Grande, UFCG., Campina Grande 58401-490, Brazil; ⊥ Materials, Electrochemistry and Polymers Laboratory (LAMEP), Federal University of Uberlândia, UFU., Ituiutaba 38304-402, Brazil

## Abstract

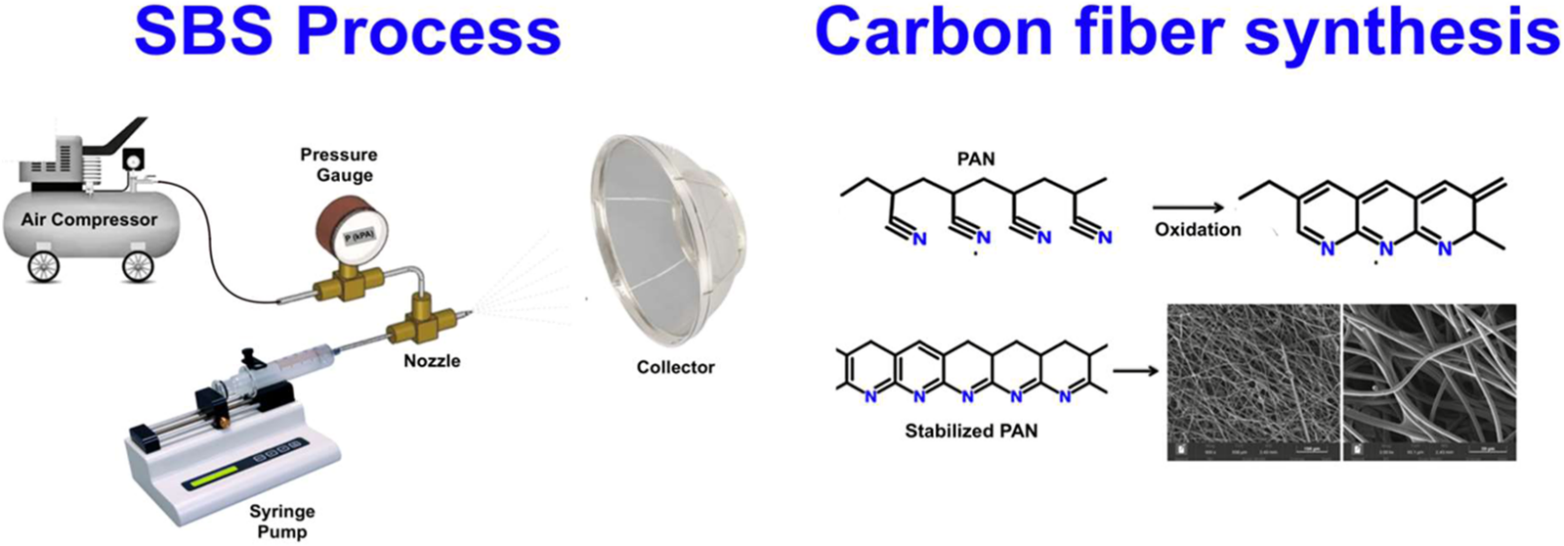

In this work, carbon fibers were produced using the solution blow spinning (SBS) technique from polyacrylonitrile (PAN) blended with 0, 2.5, 5, and 10% of poly(vinylpyrrolidone) (PVP). Spun fibers were carbonized in a tubular oven and subsequently characterized by X-ray diffraction (XRD), Fourier-transform infrared spectroscopy (FTIR), Raman spectroscopy, carbon−nitrogen elemental analysis, and scanning electron microscopy (SEM) to observe their microstructural properties. Additionally, electrochemical tests, including potentiodynamic, potentiometric, and cyclic voltammetry, were conducted to evaluate the hydrogen evolution reaction (HER). Spectroscopic characterizations indicated that carbon fibers were produced by SBS. Moreover, it was possible to control the HER to suppress hydrogen evolution in lead-acid batteries.

## Introduction

The evolution of hydrogen in the negative plate of lead/acid batteries during overcharge or float charge cycles can be a critical problem that affects the efficiency, safety, and useful life. Hydrogen evolution not only reduces battery efficiency by diverting the payload current but also can lead to water loss, contributing to the corrosion of the positive grids and general battery degradation through electrochemical processes associated with overcharging. On the other hand, during the discharge cycle, irreversible hard sulfation in the negative electrode is a critical phenomenon that occurs in lead/acid batteries at a high-rate partial state of charge duty (for example, in hybrid/electric vehicles). During discharge, lead sulfate (PbSO_4_) is produced in the negative plate, and part of the oxidized lead is recovered in the charging, making the process partially reversible for most of its useful life. If a battery is not recharged properly or is left discharged for long periods, PbSO_4_ not recovered forms crystal layers that are not glued to the lead electrode. Since these layers are difficult to dissolve, they can result in permanent sulfation. The loss of water of the electrolyte due to hydrogen evolution can significantly increase H_2_SO_4_ concentration, further sulfating the cathode plate and resulting in safety risks.^1−6^ For that reason, recent research shows that carbon-based additives such as carbon black, carbon nanotubes, graphene oxide, and graphite, among others, can be promising alternatives to reduce lead sulfation, lower HER, and improve the energy-to-weight ratio of LAB. However, it is still scarce to find works that use nano- and microcarbon fibers to improve the efficiency of lead batteries, especially those manufactured by the electrospinning technique (ES) and solution blow spinning (SBS).^7,8^

Equations 1 and 2 show the reactions involved in the discharge and charge of the negative plate, respectively. The primary reaction involves the conversion of lead (Pb) into lead sulfate (PbSO_4_) while sulfuric acid (H_2_SO_4_) is consumed at the negative plate. Equation 2 shows the recovery of lead from the negative plate during charging. At the positive plate, PbO_2_ is reduced to PbSO_4_, and water is produced during discharge as shown in eq 3. In the charging, PbO_2_ is recovered (eq 4). The density of the electrolyte decreases during discharge, which is a key indicator of the state of charge of the battery. During a lifetime, besides irreversible sulfation, batteries can experience overcharging and charge fluctuations, leading to hydrogen production in the negative plate and oxygen evolution (by water splitting) in the positive plate during a charge as shown in eqs 5 and 6.^9,10^
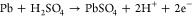
1
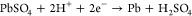
2
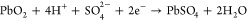
3
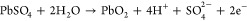
4
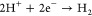
5
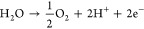
6In pursuit of mitigating sulphation in lead-acid battery negative plate during a high-rate partial state of charge duty, amounts of carbon additives larger than normally used were applied to the negative active material. Although positive results were achieved regarding sulfation, the higher amount of carbon additive increased hydrogen evolution and water loss.^11,12^ Therefore, the understanding and control of hydrogen evolution are essential to improving the performance and durability of lead/acid batteries, which are still used worldwide in automotive vehicles due to their relatively low cost and ability to deliver high peak electric currents. This topic is particularly relevant as the automotive industry seeks more efficient batteries with lower maintenance requirements for hybrid/electric vehicles. One of the means to control the hydrogen evolution reaction can be using carbon fibers on the lead surface,^13−15^ as they have also been applied to other metals.^16−18^

Carbon fibers (CFs) made from PAN are the most efficient ones because they can be converted into fibers with high percentages of carbon due to a continuous polymer backbone and ideal positioning of nitrile groups that facilitate cyclization reactions. In addition to PAN, other precursors such as poly(vinylpyrrolidone) (PVP), when mixed with PAN, modify fiber surface roughness, improving the electrical properties of devices made with such material.^19−24^ The manufacturing process of PAN-based CFs can completely change their properties and, consequently, their quality.^25^ Therefore, it is necessary to evaluate in advance which method is most suitable for a desired application. Typically, CFs are made by spinning methods, which can be divided into solution and melt spinning, with the former being subdivided into dry spinning and wet spinning. Wet spinning is the most widely used in industry due to the greater ease of adapting a plant to carry out this process, better factory control, and high molecular orientation of the fibers.^26^

More recently, with the advance of nanotechnology, other fiber spinning methods have been used to produce micro-and nanofibers, including the two most well-known techniques, viz., electrospinning (ES) and solution blow spinning (SBS).^27^ SBS has been gaining attention from the scientific community due to its simplicity, low costs, and ease of fiber production, both 2D and 3D.^28,29^ However, SBS is still very little explored by the scientific community and industry to produce carbon fibers.^28^ SBS can be used to produce CFs from a solution of dimethylformamide (DMF) with PAN and/or PVP. In this technique, the polymer is injected through a concentric nozzle system in a controlled manner, and the compressed air exiting the outer nozzle causes polymer chain orientation while the solvent evaporates across the working distance between the spinning nozzle and the collector, forming carbon fibers with micro- and nanometric diameters. Furthermore, it is possible to blend PAN with PVP to obtain fibers that can be applied in different areas, such as capacitors and batteries.^28,30^

In this study, we present, for the first time in this application, a novel approach to the fabrication of carbon fibers (CFs) incorporating up to 10% polyvinylpyrrolidone (PVP) specifically for lead-acid battery electrodes, utilizing the innovative solution blow spinning (SBS) technique. This method aims to mitigate the hydrogen evolution reaction (HER) and investigate the potential applications of these fibers in energy storage devices. Our methodology represents a significant departure from conventional techniques, such as electrospinning, by offering a more cost-effective and scalable solution for the synthesis of carbon-based electrodes. The synergistic blending of polyacrylonitrile (PAN) and PVP during the fiber manufacturing process plays a crucial role in further diminishing the HER, thereby optimizing the electrochemical performance of the electrodes for energy storage applications. Additionally, the incorporation of SBS-derived CFs contributes to the enhanced microstructural stability of the electrodes. This research represents a meaningful advancement in the field of energy storage technologies. Comprehensive characterization techniques were employed to evaluate the microstructural features of the CFs, underscoring the efficacy and versatility of this innovative fabrication approach.

## Experimental Section

**Figure 1:**
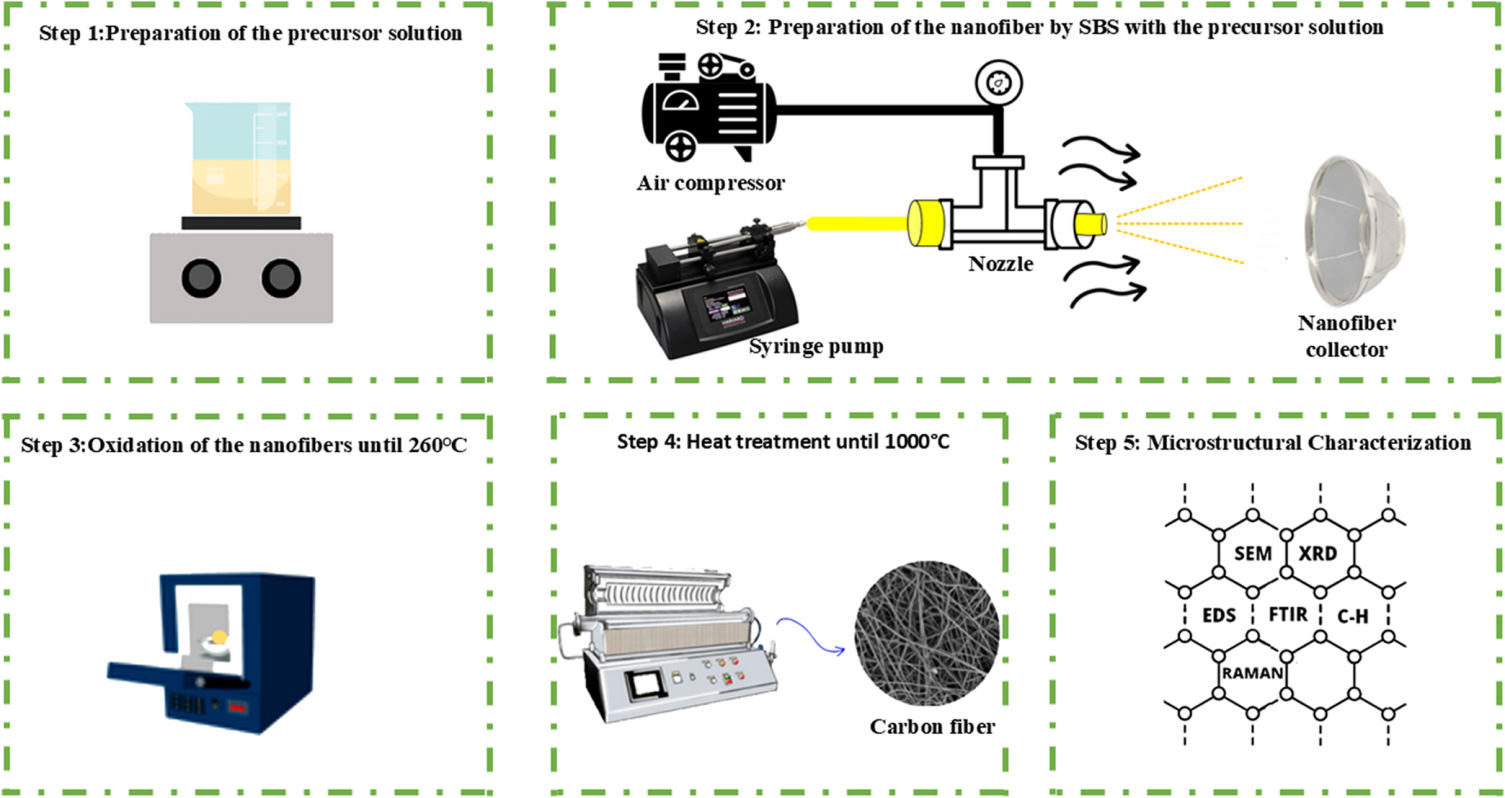
Carbon fibers manufacturing steps: (1) Preparation of the solution, (2) process of obtaining precursor fibers, (3) first firing at 260 °C, (4) second firing at 1000 °C in a nitrogen atmosphere, and (5) microstructural characterizations.

Polyacrylonitrile (PAN, *M*_W_ = 1,50,000 g mol^−1^, Quimlab-Brazil), polyvinylpyrrolidone (PVP-K90, *M*_M_ = 13,00,000 g mol^−1^, Êxodo Científica-Brazil), and dimethylformamide (DMF, 99%, Vetec) were used in the synthesis of carbon fibers, which were prepared in three stages (spinning, oxidation, and carbonization) by SBS. For the electrochemical analysis, a lead screw was used as the counter electrode and working electrode, in addition to the Hg/Hg_2_SO_4_/K_2_SO_4_(sat) reference electrode. Details about fiber spinning are found in Figure 1.

### Synthesis of Carbon Fibers

In total, four solutions were prepared. The first one was 10% w/v PAN + DMF, and from the second onward, 2.5, 5, and 10% PVP were added in relation to the initial mass of PAN and always maintaining the proportion of 10% w/v in relation to the total mass of the polymers and the total volume of the solvent. The mixture was left in a water bath at 60 °C for 6 h with moderate stirring. Then, the green fibers were produced using the following conditions: a polymer ejection rate of 3 mL h^−1^, a pressure of 0.14 MPa, and a working distance of 20 cm. These fibers were oxidized in a muffle furnace at a maximum temperature of 260 °C, using the following conditions: a rate of 3 °C min^−1^ up to 100 °C and holding for 5 h, followed by further heating at 3 °C min^−1^ to 260 °C and holding for another 5 h. After oxidation, fibers were carbonized in a tubular oven in a nitrogen atmosphere (500 mL min^−1^) at a rate of 5 °C min^−1^ up to 1000 °C and allowed to rest for 2 h. Finally, the carbon fibers produced were characterized by X-ray diffraction (XRD), Fourier-transform infrared spectroscopy (FTIR), Raman spectroscopy, carbon−nitrogen elemental analysis, and scanning electron microscopy (SEM).^31−33^ Details on the characterization of carbon fibers are found in the Supporting Information.

### Electrochemical Tests

**Figure 2:**
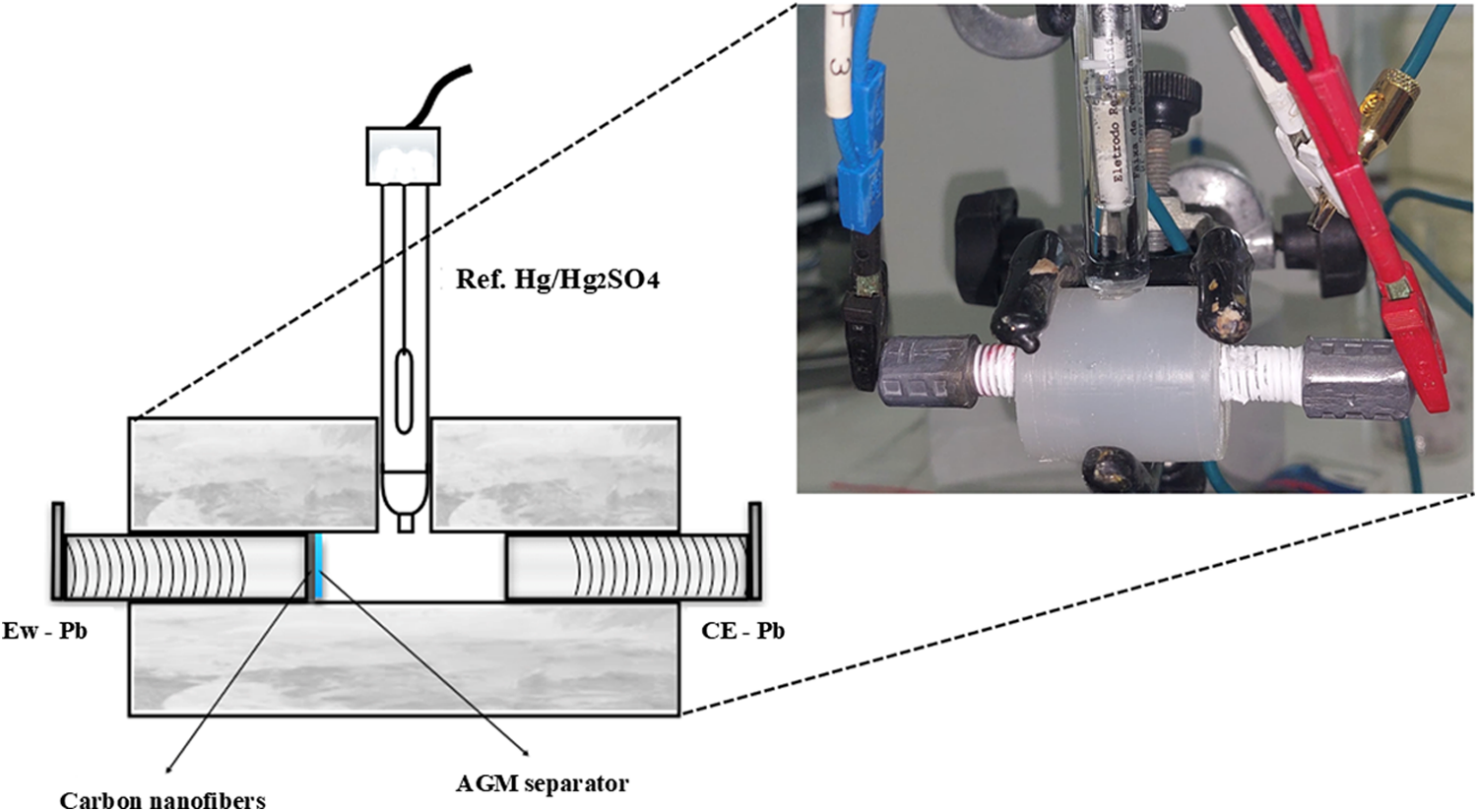
Three-electrode cell: WE (99.9% Pb-working electrode), CE (Pb-counter electrode), and RE (Hg/Hg_2_SO_4_/K_2_SO_4_(sat)). Procedure for assembling the electrochemical cell and conducting the analysis of carbon fibers deposited on a lead surface immersed in a 4.7 mol L^−1^ of H_2_SO_4_ solution.

Carbon fibers obtained from different PVP compositions were deposited on a lead alloy screw. Composite forms were evaluated for their ability to increase the potential for hydrogen production during an overcharge or float charge. Results were compared with those of the lead alloy screw without fiber. Figure 2 shows the three-electrode cell used in the electrochemical tests where the 99.9% Pb alloy was used (without and with fiber deposits) as the working electrode (E_w_), 99.9% Pb alloy as the counter electrode, both with approximately 0.72 cm^2^ of geometric surface area and a Hg/Hg_2_SO_4_/K_2_SO_4_(sat) as the reference electrode.

Initially, a −1.2 V polarization was applied for 1 h to remove oxides and sulfates from the lead alloy. All tests were carried out in triplicate at room temperature. All of the curves presented were chosen in the third replicate of each electrochemical test: linear polarization, chronopotentiometry, and cyclic voltammetry, taken as metastable conditions of the working electrode surface. All potentials were measured with Ohmic drop compensation between the reference electrode and the samples. A potentiodynamic polarization test (−1.0 to −2.0 V) was carried out on the lead alloy electrode (reference sample - blank) to choose the hydrogen reduction current for the chronopotentiometric tests, using a concentration of 4.7 mol L^−1^ of H_2_SO_4_. For the cyclic voltammetric tests (−0.5 to −1.5 V; 20 mV/s), results of the hysteresis of charge (cathodic peak) and discharge (anodic peak) currents were evaluated for the lead samples deposited with different concentrations of carbon fibers and compared to the lead alloy alone.^34−36^

## Results and Discussion

**Figure 3:**
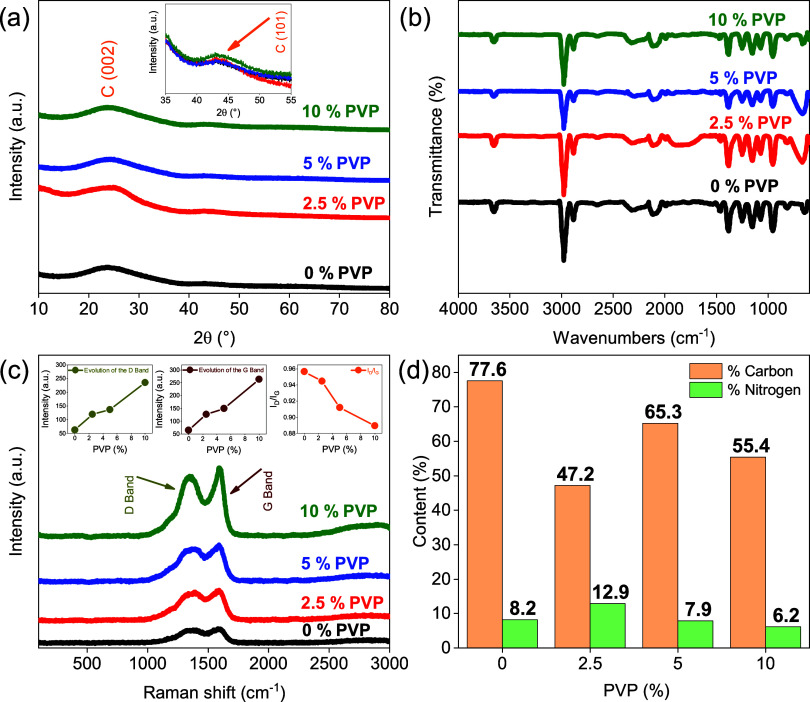
(a) XRD, (b) FTIR, (c) Raman, and (d) elemental composition of carbon and nitrogen of the fibers obtained by solution blow spinning.

Figure 3 shows the X-ray diffraction patterns of pure PAN and the blend with PVP additions after carbonization. All peaks are characteristic of a predominantly amorphous structure but with a slightly hard carbon structure.^37^ The peaks located at 2θ = 24.28 and 43.69° correspond to the (002) and (101) planes of the graphene layers, which together with the following analyses will prove the existence of CFs.^38^ The FTIR spectra shown in Figure 3b draw attention to three peaks at 815, 2115 cm^−1^, and in the region of 3200−3700 cm^−1^, which are due to the presence of aromatic carbons, formation of isocyanate, and O−H, respectively. This may indicate that there was a cyclization reaction during the carbonization process, confirming the presence of CFs. Another indication is that the PAN polymer as a precursor does not have peaks in the 815 cm^−1^ regions, and there is a peak at 2244 cm^−1^, which refers to the nitrile group C≡N that gave rise to the peak at 2115 cm^−1^.^37,39^Figure 3c shows the Raman spectra where two bands located at ∼1330 cm^−1^ (D band) and ∼1580 cm^−1^ (G band) are observed. The D band is related to defects and disordered carbon structures, while the G band confirms that there is a graphitic structure in part of the carbon fibers or simply a hard carbon structure in this case. The degree of crystallinity of carbon-based materials can be obtained from the ratio between the intensity of the D band (I_D_) and the intensity of the G band (I_G_), that is, I_D_/I_G_. In this research, the increase in PVP content causes a decrease in the I_D_/I_G_ ratio, indicating that there is more regularity of the graphene layers in the structure.^40,41^ The elemental compositions of carbon and nitrogen are shown in Figure 3d. As observed, the contribution of carbon varies in the range of 47.2−77.6%, while nitrogen varies from 6.2 to 12.9%. The remainder of the compositional range refers to oxygen because of the temperature of carbonization that was only until 1000 °C.^26,42^

**Figure 4:**
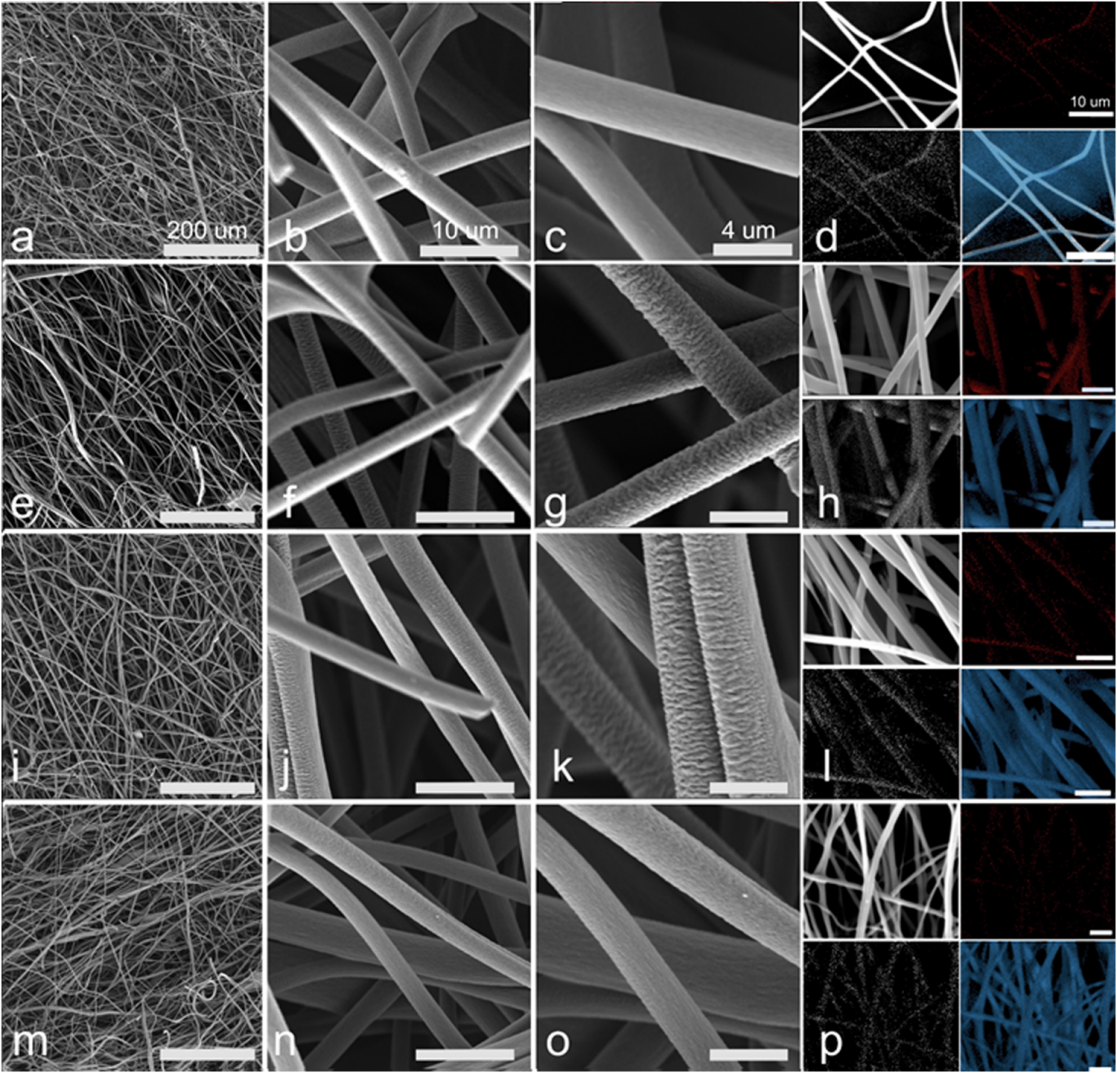
SEM images accompanied by chemical mapping of carbon fibers obtained by solution blow spinning: (a–d) 0, (e–h) 2.5, (i–l) 5, and (m–p) 10% PVP.

Figures S1a–c show the transformation of fibers at each stage of the process. Figure 4 shows SEM images of carbon fibers as a function of the PVP content. In the sample with 0% PVP (Figure 4a–d), the fiber surface is practically smooth and free of defects and porosity, while under the conditions with 2.5% (Figure 4b–h) 5% (Figure 4i–l) and 10% (Figure 4m–p) PVP, it is possible to notice the presence of rough fibers. Furthermore, the length of the fibers exceeds 100 μm. The fiber diameter distribution curves are shown in Figure S2. As observed, the average diameters were estimated at 2.2, 2.4, 2.8, and 2.5 μm for 0, 2.5, 5, and 10% PVP, respectively. Added to this, chemical mapping (see Figure 4) reveals a uniform distribution of the elements carbon (blue) and nitrogen (red).^37,43−45^

### Electrochemical Tests

**Figure 5:**
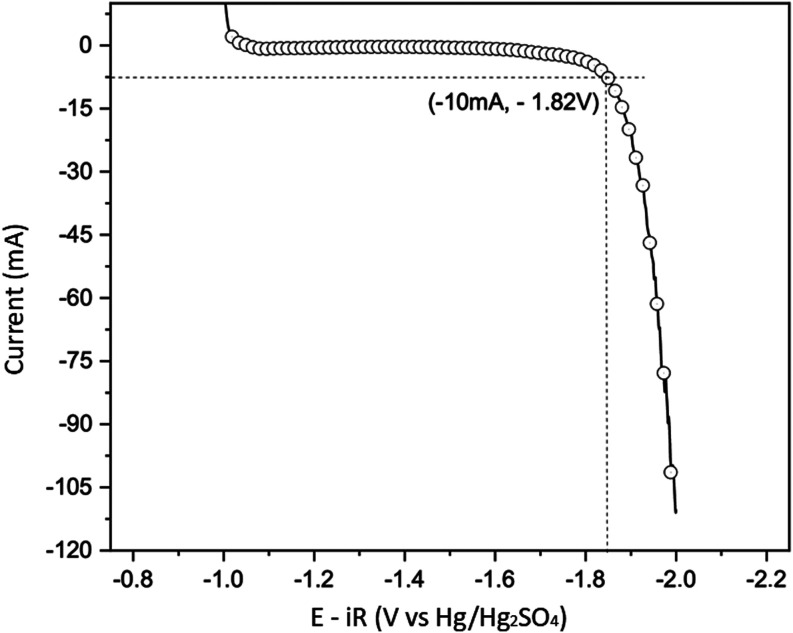
Potentiodynamic curve of 99.9% Pb alloy/H_2_SO_4_ 4.7 mol L^−1^ system. Temperature: 25 ± 1 °C. Reference electrode: Hg/Hg_2_SO_4_/K_2_SO_4_(sat). Initial potential: −1.0 V. Final potential: −2.0 V. Scan rate: 20 mV s^−1^. Potentiostatic step before the potentiodynamic test: −1.2 V for 1 h.

**Figure 6:**
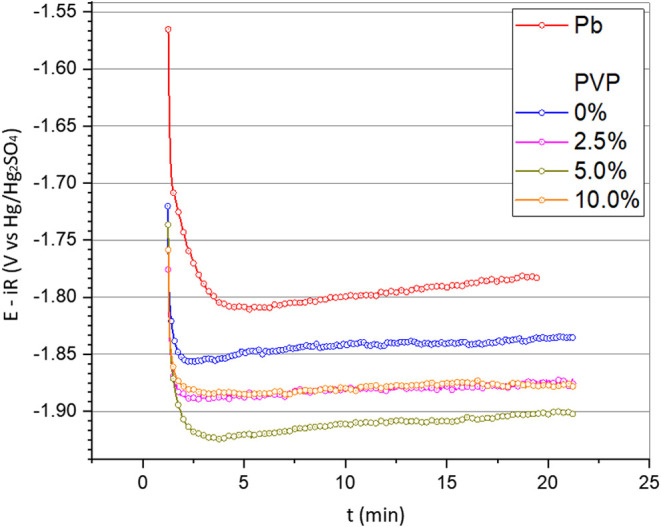
Potential variation from potentiometric tests to a constant current intensity of −10.0 mA. Temperature: 25 ± 1 °C. System: Pb/film with different PVP contents/H_2_SO^4^ 4.7 mol L^−1^. Reference electrode: Hg/Hg_2_SO_4_/K_2_SO_4_(sat). Potentiostatic step: −1.2 V for 1 h. For comparison, potentiometry of the Pb/H_2_SO_4_ 4.7 mol L^−1^ system was also performed under the same conditions.

**Figure 7:**
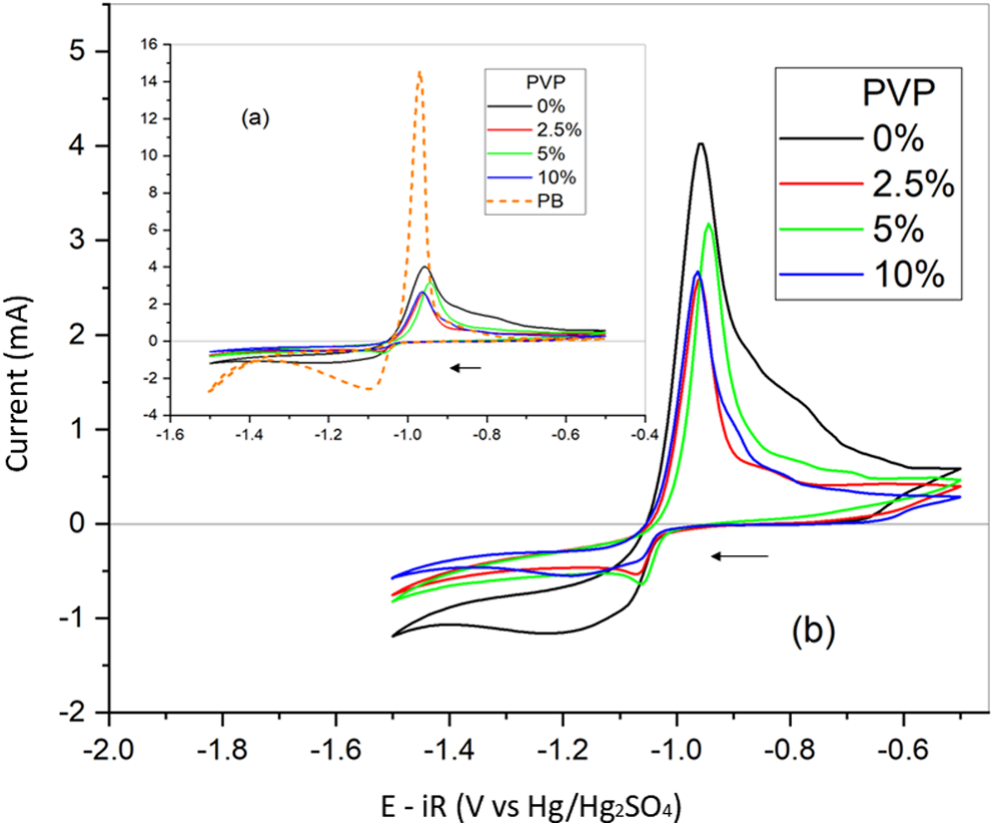
Cyclic voltammetry results for all samples used, Pb taken as reference. (a) Pb + fibers. (b) fibers. Temperature: 25 ± 10 °C. System: Pb/film with different PVP contents/H_2_SO_4_ 4.7 mol L^−1^. Reference electrode: Hg/Hg_2_SO_4_/K_2_SO_4_(sat). Scan rate: 20 mV s^−1^. Potentiostatic step previous to −1.2 V for 1 h.

Figures 5, 6 and 7a,b show the results obtained for the electrochemical tests of linear polarization, potentiometry, and cyclic voltammetry, respectively. The first linear polarization test aims to determine the current intensity associated with a small hydrogen production simulating a high-rate charge, an overcharge, or a float charge situation in lead-acid battery operation. Figure 5 shows the linear polarization results for the 99.9% Pb alloy Hg/Hg_2_SO_4_/K_2_SO_4_(sat) taken as a reference. A cathodic potentiostatic step was done before the potentiodynamic sweep to reduce possible PbSO_4_ and lead oxides formed on the lead alloy surface.^46^ This was also done in the other two electrochemical tests. The current intensity of −10 mA was chosen at the inflection point of the curve for a potential of approximately −1.82 V; higher values could interfere with the measurements of the subsequent comparative potentiometry tests due to the great production of hydrogen bubbles. Figure 6 shows the results of the potentiometric tests carried out for the different compositions of carbon fibers deposited on the lead alloy. After 20 min, the layer formed only with the PAN (or 0% PVP) precursor showed a slightly higher overpotential of around −1.86 V, indicating a small increase of resistance due to the deposited layer producing the same amount of hydrogen as the lead alloy electrode. On the other hand, the addition of PVP showed that the 5% composition performed better, requiring an overpotential of around −1.92 V to produce the same amount of H_2_. This result, compared to the other compositions used with 2.5 and 10% PVP, can be explained by a better distribution of fibers, resulting in a higher coverage due to the greater roughness of the fibers, which promotes greater contact and anchoring points (Figure 4 of SEM (i-l)). Looking at Figure 3 of the article (image D), the 5% PVP composition with 65.3% carbon and 7.9% nitrogen may represent an ideal composition to suppress the hydrogen evolution when compared to the others.^18,46−49^

Figure 7a,b shows the results of the voltammetric curves obtained from all samples evaluated. The cyclic voltammetry (*C–V*), in this case, simulates the conditions for the discharge/charge cycle of a lead-acid battery negative electrode. The oxidation of Pb to PbSO_4_ (discharge) happens in the anodic sweep, while in the cathodic sweep, the reduction of PbSO_4_ to Pb (charge) occurs.^5,50^Figure 7a shows that there is a great decrease of the anodic current peak when carbon fibers are deposited on the lead alloy electrode surface. This means less formation of PbSO_4_, which is due to the hindering effect of carbon fibers on the surface of the lead alloy. Figure 7a,b also shows that for all of the lead alloy samples, the cathodic current peak is smaller than the anodic current peak. This means that not all of the PbSO_4_ formed in the discharge is recovered in the charge. The interpretation of these results is that during the charge disruption, the PbSO_4_ occurs. Only the PbSO_4_ attached to the lead alloy surface is recovered.^5,51−53^

Although the presence of the coating produces a significant decrease in the PbSO_4_ formation, considering the aspect of hydrogen production during the overcharge cycle and comparing with the potentiometric data obtained (Figure 6), the fiber composition with 5% PVP shows the best results.

## Conclusions

Carbon fibers based on PAN + PVP were produced by using the solution blow spinning technique. XRD showed the characteristic amorphous structure of carbon fibers. SEM images confirmed the fibrillar aspect of these spun fibers, and the Raman analysis demonstrated the degree of graphitization after carbonization, showing the formation of carbon fibers. Moreover, results show that lead samples coated with carbon fibers contributed to a reduction in hydrogen evolution. In this specific case, the sample with 5% PVP showed the best result in a situation where overcharge is involved, implying that carbon fibers can be potentially used to improve lead/acid batteries.

## Supporting Information Available

**Figure S1.** Fibers (a) before oxidation, (b) oxidized and (c) carbonized; **Figure S2.** Diameters distribution histogram of carbon fibers. (PDF)
