# Supplementary material for: Carbon Fibers from PAN/PVP Blends by Solution Blow Spinning to Suppress Hydrogen Evolution in Lead-Acid Batteries
Source: ACS Omega. 2025 Apr 22;10(17):17353–60. doi: 10.1021/acsomega.4c10531 (PMC12059894; doi:10.1021/acsomega.4c10531)
Supplement: Supplementary file 1 — ao4c10531_si_001.pdf [file ao4c10531_si_001.pdf]

Carbon fibers from PAN/PVP blends by solution blow spinning to suppress hydrogen evolution in lead-acid batteries

Caio M. S. Lopes [a,b], *Juan P. S. Cruz* [a,b], *Rafael A. Raimundo* [a,c], *Vinícius D. Silva* [a,b], *Rogério T. Ribeiro* [a], *Daniel A. Macedo* [a], *Eudésio O. Vilar* [d], *Gilberto A. O. Brito* [e], *Eliton S. Medeiros* [a,b*]

[a] Department of Materials Science and Engineering, UFPB, 58051-900, João Pessoa, Brazil

[b] Materials and Biosystems Laboratory (LAMAB), DEMAT, UFPB, 58051-900, João Pessoa, Brazil

[c] TEMA - Centre for Mechanical Technology and Automation, Department of Mechanical Engineering, University of Aveiro, 3810-193, Aveiro, Portugal

[d] Electrochemical Eng. Laboratory (LEEQ), Federal University of Campina Grande, UFCG. 58401-490 Campina Grande, Brazil

[e] Materials, Electrochemistry and Polymers Laboratory (LAMEP), Federal University of Uberlândia, UFU. 38304-402 Ituiutaba, Brazil

[*] Corresponding author: esm@academico.ufpb.br

A. Information on the Morphological and structural characterization of carbon fibers

The structure of the carbon fibers was characterized by X-ray diffraction (RIGAKU diffractometer, Miniflex II) equipped with Cu-Ka radiation (k = 1.5418 Å). Diffraction patterns were obtained in the angular range of 10-80°, with a step size of 0.02° and an acquisition time of 1 s per step. Fourier transform infrared spectroscopy (FTIR) measurements (Shimadzu IRPrestige21 spectrophotometer) were obtained in the range of 400 and 4000 cm^{-1}, using pressed KBr pellets containing 1% by weight of sample. Raman spectroscopy measurements were performed at room temperature using a 532 nm laser as an excitation source (LabRAM-HR Evolution-HORIBA) with a laser power was maintained at 1 mW with an acquisition time of 10 s for accumulation. The percentages of carbon and nitrogen were determined using the elemental analyzer (CHN268, LECO), with EDTA standard, operating at 950 °C. Morphological characterization was carried out using scanning electron microscopy (SEM, TESCAN, model VEGA4 LMS) equipped with an EDS Essence model, 30 mm^2 (WD=5.77 and 6.08 mm, voltage of 15 kV).

B. Information on microstructure of carbon fibers

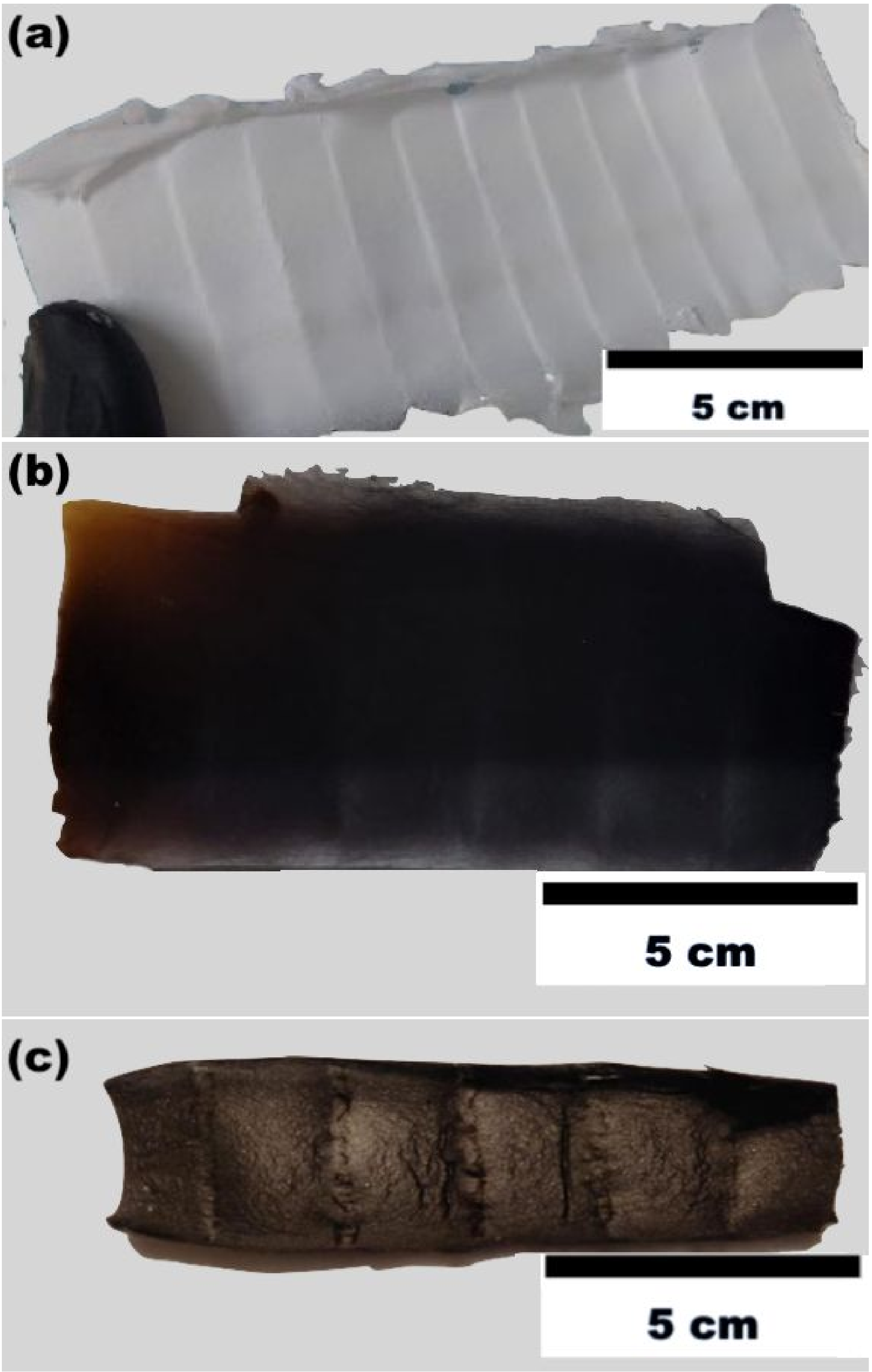

Figure S1. Fibers (a) before oxidation, (b) oxidized and (c) carbonized.

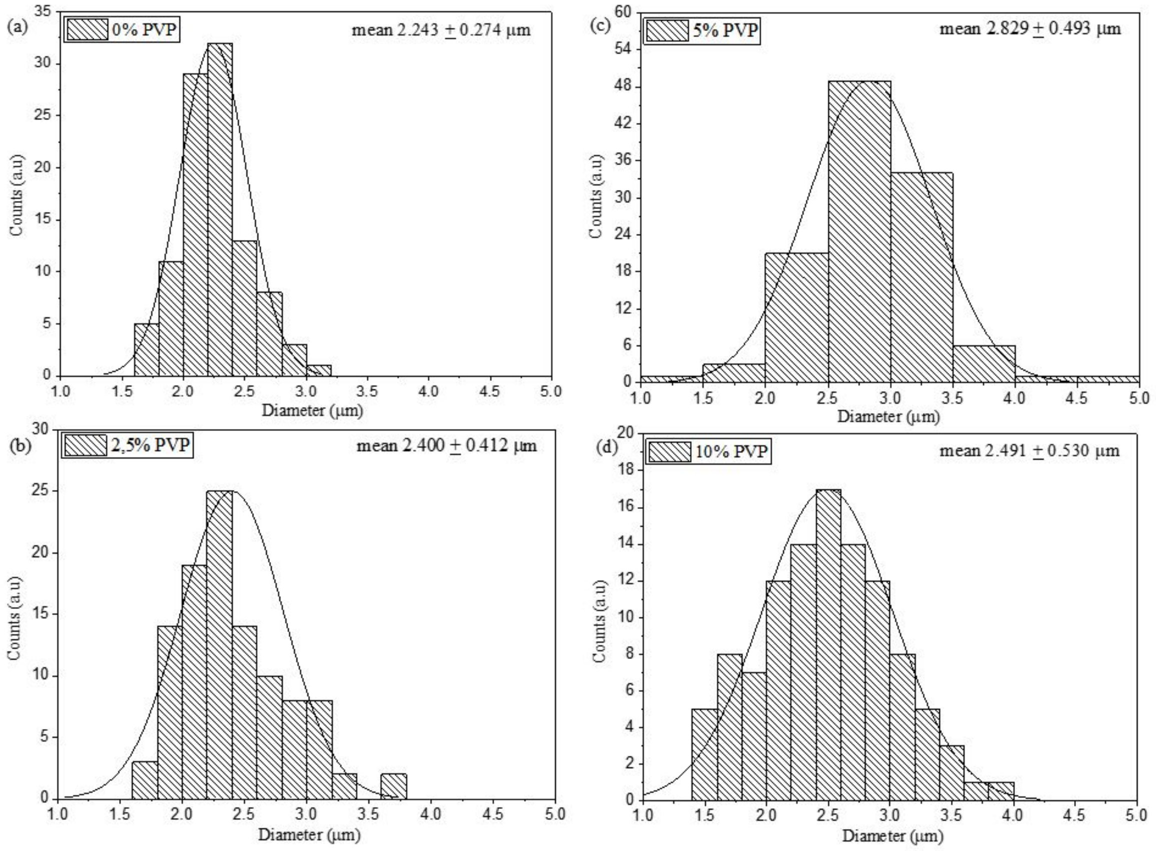

Figure S2. Diameters distribution histogram of carbon fibers.
